# Impact of oxytocin discontinuation on fetal heart rate and uterine contractility: A pre‐specified ancillary analysis embedded within a randomized trial

**DOI:** 10.1111/aogs.70295

**Published:** 2026-07-17

**Authors:** Lise Lafforgue, Aude Girault, Loïc Sentilhes, Raoul Desbriere, Diane Korb, Tiphaine Barjat, Camille Le Ray, Charles Garabedian

**Affiliations:** ^1^ Department of Obstetrics CHU Lille Lille France; ^2^ Department of Obstetrics and Gynecology Port‐Royal Maternity Hospital, AP‐HP, Cochin Hospital, FHU PREMA Paris France; ^3^ Obstetrical Perinatal and Pediatric Lifelong Epidemiology Research Team (OPPaLE), Center for Research on Epidemiology and Statistics (CRESS) U1153 Université Paris Cité, Institut Santé des Femmes, INSERM, INRAE Paris France; ^4^ Department of Obstetrics and Gynecology Bordeaux University Hospital Bordeaux France; ^5^ Department of Obstetrics and Gynecology Saint Joseph Hospital Marseille France; ^6^ Department of Obstetrics and Gynecology Robert Debré Hospital, AP‐HP Paris France; ^7^ Department of Obstetrics and Gynecology Saint Etienne University Hospital Saint‐Etienne France; ^8^ University of Lille, ULR2694 – Metrics Lille France

**Keywords:** fetal heart rate, hypoxia, labor, oxytocin, uterine contractility

## Abstract

**Introduction:**

Oxytocin is widely used to augment uterine contractions during labor. However, its use has been associated with fetal heart rate (FHR) abnormalities and neonatal morbidity, which may be reduced by discontinuing oxytocin during labor. We aimed to assess the impact of oxytocin discontinuation at the onset of the active phase of labor on FHR patterns and uterine contractility.

**Material and Methods:**

This study is a pre‐specified ancillary analysis of the STOPOXY trial, a multicenter, randomized, open‐label, controlled superiority trial conducted in 21 French maternity units between January 2020 and January 2022, which aimed to assess the impact of oxytocin discontinuation during active labor on neonatal morbidity. Participants who received oxytocin before 4 cm dilation were randomly assigned (1:1) to either oxytocin discontinuation or oxytocin continuation. For the present analysis, we included women from the per‐protocol discontinuation group of the parent trial. Inclusion was restricted to the six centers with electronic cardiotocography storage where valid cardiotocography recordings were available for at least 1 h before and 1 h after oxytocin discontinuation. Using a paired before‐and‐after design, FHR parameters (classified according to FIGO criteria) and uterine activity were compared during the 60 min preceding versus the 60 min following oxytocin discontinuation by independent obstetricians blinded to neonatal outcomes. Changes in FHR pattern were categorized as no change, improvement, or deterioration.

**Results:**

284 women fulfilled the eligibility criteria. Following oxytocin discontinuation, mean FHR increased (135 vs. 137.5 bpm; *p* < 0.002) and FHR variability significantly changed (*p* = 0.010), with a lower rate of reduced variability (3.9% vs. 2.5%) and a higher rate of normal variability (48.2% vs. 53.3%). The proportion of tracings with decelerations significantly decreased (64.1% vs. 48.6%; *p* < 0.001). Uterine activity decreased, with fewer uterine contractions (4.0 vs. 3.5 contractions per 10 min; *p* < 0.001).

**Conclusions:**

Among women receiving oxytocin during early labor, discontinuation at the onset of the active phase was associated with improved FHR patterns and reduced uterine activity, suggesting a lower fetal stress and tachysystole. Further studies are needed to assess whether these changes affect labor management or maternal experience.

## Graphical abstract

The impact of oxytocin discontinuation at the onset of the active phase of labor on fetal heart rate (FHR) patterns and uterine activity; One hour following discontinuation, the mean FHR baseline slightly increased, FHR patterns improved—particularly with reduced decelerations and improved variability—and uterine activity decreased. Moreover, among the 184 women with initially abnormal tracings, 35.3% normalized within this hour, which was associated with a significant reduction in composite neonatal morbidity.

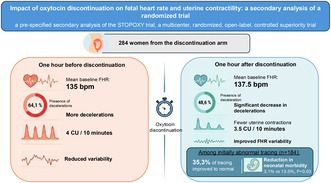

**Abbreviations**
CI: confidence intervals
CTG: cardiotocography
FHR: fetal heart rate
FSpO_2_
: fetal oxygen saturation
IQR: interquartile ranges
OR: odds ratio
UC: uterine contraction

**Key message**

Discontinuing oxytocin at the onset of the active phase of labor significantly improves fetal heart rate patterns and reduces uterine contractility.

## 1 INTRODUCTION

Synthetic oxytocin is widely used to augment uterine contractions (UC) during labor by increasing their frequency and intensity. However, its use has been associated with adverse neonatal outcomes. In particular, oxytocin administration is associated with an increased risk of uterine tachysystole and fetal heart rate (FHR) abnormalities, which may compromise fetal oxygen and increase neonatal morbidity, including neonatal acidosis.^1^, ^2^, ^3^

These associations are thought to result from excessively short intervals between uterine contractions, which limit adequate fetal reoxygenation.^3^ Previous studies have shown that the nadir in oxygen saturation occurs approximately 90 s after the peak of the contraction, and that a similar duration is required to return to baseline levels.^4^ By shortening the interval between uterine contractions, oxytocin may therefore induce a progressive decline in fetal oxygen saturation (FSpO_2_) with recovery often observed only after oxytocin discontinuation.^3^, ^5^

Despite these concerns, there is currently no clear consensus regarding oxytocin management once the active phase of labor is established. Several randomized trials have evaluated dose reduction strategies, including discontinuation of oxytocin infusion at the onset of the active phase, with the aim of minimizing maternal and neonatal adverse effects. In a meta‐analysis of 15 studies, including two major trials,^6^, ^7^ Whitley et al. demonstrated that discontinuing oxytocin at the onset of the active phase significantly reduced the risk of tachysystole (RR = 0.45; 95% CI, 0.34–0.60) and FHR abnormalities (RR = 0.64; 95% CI, 0.49–0.82).^8^ However, none of the included studies provided a detailed assessment of the specific impact of oxytocin discontinuation on FHR patterns and uterine contractility.

Therefore, the objective of this study was to assess the impact of oxytocin discontinuation at the onset of the active phase of labor on fetal heart rate patterns and uterine contractility.

## 2 MATERIAL AND METHODS

### 2.1 Study design

This study is a pre‐specified ancillary analysis of the STOPOXY trial, a multicenter, randomized, controlled trial conducted across 21 maternity units in France between January 2020 and January 2022.^7^, ^9^ The primary objective of the parent trial was to assess the impact of oxytocin discontinuation during the active phase of labor on neonatal morbidity. For this pre‐specified ancillary analysis, FHR tracings were prospectively collected in six centers. These recordings were requested for centers with electronic cardiotocography (CTG) storage. Thus, CTG data were retrieved from six participating centers: Lille, Paris (Port Royal and Robert Debré), Bordeaux, Saint‐Etienne, and Marseille.

Participants who received oxytocin before 4 cm of cervical dilation were randomized in a 1:1 ratio to either an oxytocin discontinuation group (infusion stopped beyond a cervical dilation equal to or greater than 6 cm) or a continuation group (infusion maintained until delivery). Randomization was stratified by center and parity. The primary outcome of the STOPOXY trial was neonatal morbidity assessed at birth using a composite variable defined as: umbilical arterial pH <7.10, base excess >10 mmol/L, umbilical arterial lactates >7 mmol/L, 5‐min Apgar score <7, or admission to the neonatal intensive care unit. No significant difference in the primary outcome was observed between the two groups.

### 2.2 Population and sampling

Eligibility criteria for the original STOPOXY trial included women aged ≥18 years with a singleton term pregnancy (≥37 weeks) and a fetus in cephalic presentation receiving oxytocin before 4 cm cervical dilation. Women with a scarred uterus, estimated fetal weight <3rd centile, known congenital anomalies, or abnormal fetal heart rate at randomization were excluded.

For the present analysis, we included only women who delivered in one of the six maternity units where fetal heart rate tracings were systematically collected as part of the study protocol. This analysis included women from the per‐protocol population of the discontinuation arm who had valid CTG recordings available for at least the hour preceding and the hour following discontinuation. We additionally excluded participants with uninterpretable FHR tracings (poor quality of signal) and those who delivered within 1 h following oxytocin discontinuation.

### 2.3 Analysis strategy

The study followed a paired before‐and‐after design centered on oxytocin discontinuation. FHR parameters and uterine activity were analyzed by two independent obstetricians blinded to neonatal outcomes. To define the ‘before’ and ‘after’ periods, evaluators manually identified the exact timestamp of oxytocin cessation. Consequently, they were not blinded to the exposure, and the two consecutive 60‐min windows were analyzed sequentially for each participant. Uterine activity was quantified as the mean frequency of contractions per 10 min. This design allowed each participant to serve as her own control, minimizing between‐subject variability.

FHR characteristics were extracted according to the FIGO classification^10^ and included baseline heart rate (beats per minute), variability (<5, 5–10, 10–25, or >25 bpm), presence and type of decelerations (none, early, variable, late, or prolonged), and presence/number of accelerations. Uterine activity was quantified as the mean number of contractions per 10 min averaged over the one‐hour window. A normal FHR tracing was defined as a baseline between 110 and 160 bpm, moderate variability (5–25 bpm), and no decelerations.^10^ Based on these criteria, changes in FHR pattern following oxytocin discontinuation were categorized as unchanged normal, unchanged abnormal, deterioration (transition from normal to abnormal), or improvement (transition from abnormal to normal). For additional analyses, a binary outcome was derived to compare improvement versus persistent abnormal FHR patterns. To evaluate the clinical relevance and prognostic value of FHR changes, a pre‐specified targeted analysis was performed within the subgroup of women from the discontinuation arm with an initially abnormal CTG pattern (*n* = 184). We compared labor characteristics, mode of delivery, and neonatal outcomes between women whose FHR normalized (’improvement’ group) and those whose FHR remained abnormal (’persistent abnormal pattern’ group) following oxytocin discontinuation. This analysis aimed to determine whether the observed physiological FHR improvement translated into a tangible clinical and neonatal benefit.

No core outcome set specific to intra‐partum fetal rate assessment or oxytocin management was available at the time this study was designed. Outcomes were therefore selected based on established international definitions and clinical relevance, including FIGO fetal heart rate classification and standard measures of uterine activity.

Maternal and labor characteristics—including age, pre‐pregnancy body mass index, smoking status, parity, hypertensive or diabetic disorders, mode of labor onset, epidural analgesia, cervical dilation at discontinuation, and fetal presentation—were compared between women with and without FHR improvement. Neonatal outcome was evaluated through the composite variable defined in the STOPOXY study and previously described. Missing data were excluded from the corresponding analyses (complete case approach).

### 2.4 Statistical analysis

Continuous and ordinal variables are presented as medians with interquartile ranges (IQR) and categorical variables as numbers and percentages. Paired comparisons between the pre‐ and post‐discontinuation periods were performed using the Wilcoxon signed‐rank test for continuous and ordinal variables and McNemar's test for categorical variables. Unpaired comparisons between subgroups (e.g., improvement vs. persistent abnormal FHR) were performed using the *χ* ^2^ test or Fisher's exact test (when expected cell counts were <5) for categorical variables and the Wilcoxon rank‐sum test (Mann–Whitney *U* test) for continuous variables. Exploratory analyses of maternal and labor factors associated with FHR improvement were performed using logistic regression models, including relevant clinical covariates. Neonatal outcomes were compared according to FHR evolution following oxytocin discontinuation (improvement vs. persistent abnormal tracing). Given the exploratory nature of these analyses and the limited number of adverse neonatal events, results were interpreted descriptively, and no formal adjustment for multiple comparisons was applied.

Sensitivity analyses accounting for center‐level clustering were performed using mixed‐effects logistic regression with random intercepts for study centers. Results are presented as odds ratios (OR) with 95% confidence intervals (CI). All tests were two‐sided with a significance threshold of *p* < 0.05. No adjustment for multiple testing was applied, as analyses were considered exploratory. All analyses were performed with Stata, version 15 (StataCorp, College Station, TX, USA).

### 2.5 Patient and public involvement

Patients and the public were not involved in the design, conduct, analysis, or reporting of this secondary analysis. The parent STOPOXY trial did not include formal patient or public involvement in outcome selection.

## 3 RESULTS

**FIGURE 1:**
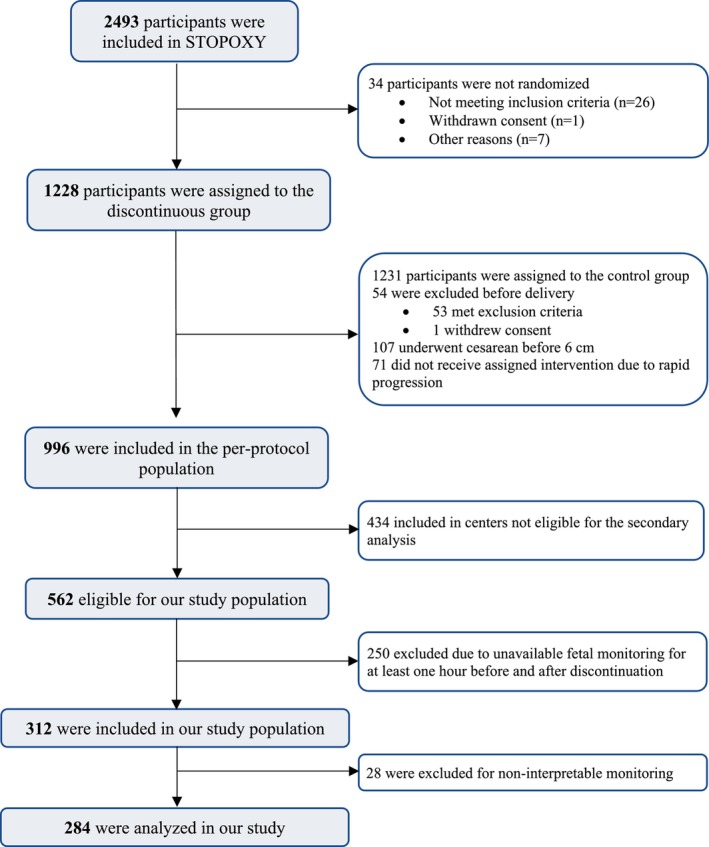
Flow chart.

Of the 996 participants in the STOPOXY per‐protocol discontinuation group, 562 delivered in one of the six maternity units where fetal heart rate tracings were prospectively collected. Among those, 312 had an available monitoring for at least 1 h before and after discontinuation and were included. Twenty‐eight women were subsequently excluded due to uninterpretable CTG recordings, resulting in a final sample of 284 women (Figure 1).

Baseline characteristics of the study population are detailed in Table 1. The median body mass index was 23.2 kg/m^2^ (IQR 20.3–27.0), and 111 women (39.1%) were multiparous. Labor was induced in 215 patients (75.7%), and 99.3% received epidural analgesia. At the time of oxytocin discontinuation, the median cervical dilation was 7 cm (IQR 6–8).

**TABLE 1:** Baseline and pregnancy characteristics.

Discontinuous oxytocin group (*n* = 284)		
Individual characteristics		
Maternal age, years	31	(28; 35)
Pre‐pregnancy BMI, kg/m^2^	23.2	(20.3; 27.0)
Smoking during pregnancy	21	(7.4%)
Multiparous	111	(39.1%)
Pre‐existing pathology		
Chronic hypertension	5	(1.8%)
Diabetes	1	(0.3%)
Pathology during pregnancy		
Hypertensive disorder (gestational hypertension or pre‐eclampsia)	10	(3.5%)
Gestational diabetes	42	(14.8%)
Labor onset		
Induction of labor	215	(75.7%)
Indication for labor induction		
Ruptured membranes	22	(10.2%)
Postdate pregnancy	34	(15.8%)
Suspected macrosomia	48	(22.3%)
Maternal pathology	18	(8.4%)
Maternal request	55	(25.6%)
Other indications^a^	38	(17.7%)
First method of induction		
Amniotomy	9	(4.2%)
Oxytocin	115	(53.5%)
Dinoprostone vaginal slow‐release system	32	(14.9%)
Misoprostol	18	(8.4%)
Ballon catheter	25	(11.6%)
Prepidil or prostin gel	16	(7.4%)
Epidural analgesia	282	(99.3%)
Time from oxytocin onset to active first stage diagnosis, min	282	(192.5; 392.5)
Cervical dilation at oxytocin discontinuation, cm	7	(6; 8)
Posterior or transverse presentation at active labor diagnosis	97	(27.5%)

*Note*: Data are *n*/*N* (%) or median (IQR).

^a^ Oligohydramnios, reduced fetal movements, fetus growth below the tenth percentile and above the third percentile for gestational age, late‐pregnancy bleeding, or maternal anxiety.

Table 2 details the changes in FHR parameters and uterine activity. Following discontinuation, the baseline FHR significantly increased (135 vs. 137.5 bpm; *p* < 0.002). A significant shift in variability was observed (*p* = 0.010), with a decrease in reduced variability (3.9% vs. 2.5%) and an increase in normal variability (48.2% vs. 53.5%). The proportion of tracing with decelerations decreased significantly (64.1% vs. 48.6%; *p* < 0.001) while no significant change was observed in the presence or number of accelerations. Uterine contraction frequency decreased from 4.0 to 3.5 per 10 min (*p* < 0.001).

**TABLE 2:** Fetal heart rate characteristics and uterine activity 1 h before and 1 h after oxytocin discontinuation.

	One hour before discontinuation	One hour after discontinuation	*p*
	*N* = 284	*N* = 284	
Overall CTG classification			
Normal	100 (35.2%)	138 (48.6%)	
Abnormal	184 (64.8%)	146 (51.4%)	
Evolution after discontinuation			
Improved (abnormal to normal)		65 (22.9%)	
Unchanged		192 (67.6%)	
Deteriorated (normal to abnormal)		27 (9.5%)	
Detailed FHR parameters			
Baseline, beats per minute (bpm) (*n* = 338)	135 (130; 142.5)	137.5 (130; 142.5)	<0.002
Tachycardia (>160 bpm)	4 (1.4)	6 (2.1)	0.32
Instability of baseline (*N* = 339)	1 (0.3)	1 (0.3)	NA
Variability			
<5 bpm	11 (3.9)	7 (2.5)	0.010
5–10 bpm	136 (47.9)	125 (44.0)	
10–25 bpm	137 (48.2)	152 (53.5)	
>25bpm	0 (0.0)	1 (0.3)	
Decelerations			
Presence of decelerations	182 (64.1)	138 (48.6)	<0.001
Number of decelerations (IQR)	1 (0; 4)	0 (0; 2)	<0.001
Type of decelerations (*N* = 338)			<0.001
None	102 (35.9)	146 (51.4)	
Early	7 (2.5)	7 (2.5)	
Variable	119 (41.9)	98 (34.5)	
Late	31 (10.9)	12 (4.2)	
Prolonged	25 (8.8)	21 (7.4)	
Accelerations			
Presence of accelerations	214 (75.3)	223 (78.5)	0.30
Number of accelerations (IQR)	2 (1; 6)	2.5 (1; 35)	0.60
Contractions (*N* = 227 in each group)			
Number of contractions	24 (22; 27)	21 (18; 25)	<0.001
Number of contractions per 10 minutes	4 (3.7; 4.5)	3.5 (3; 4.2)	<0.001

*Note*: Data are *n*/*N* (%) or median (IQR).

To account for the baseline distribution of FHR patterns, changes following oxytocin discontinuation were analyzed using a transition‐based approach rather than overall proportions. Among the 284 included tracings, 192 (67.6%) remained unchanged following oxytocin discontinuation, 65 (22.9%) improved, and 27 (9.5%) deteriorated. Specifically, among the 184 women with initially abnormal tracings, an improvement (normalization) occurred in 65 (35.3%), whereas 119 (64.7%) remained unchanged (persistently abnormal). Among the 100 women with initially normal tracings, 73 (73.0%) remained unchanged (persistently normal), while a deterioration to an abnormal pattern was observed in 27 (27.0%).

Comparisons of maternal and labor characteristics between women with and without FHR improvement did not significantly differ, except for the fetal presentation at active labor (Table 3). Posterior or transverse fetal presentation at the diagnosis of active labor was significantly more frequent among participants with FHR improvement than among those with persistent abnormal tracings (39.1% vs. 19.5%; *p* = 0.008). Regarding neonatal outcomes among the 184 women with initially abnormal tracings, FHR normalization following oxytocin discontinuation was associated with a significantly lower rate of composite adverse neonatal outcome compared with persistent abnormal tracings (3.1% vs. 13.4%; *p* = 0.035) (Table 3).

**TABLE 3:** Maternal characteristics and neonatal outcomes among women with initially abnormal FHR patterns (*n* = 184) according to their evolution after oxytocin discontinuation.

	Persistent abnormal	Improvement	*p*
	*N* = 119	*N* = 65	
Individual characteristics			
Maternal age, years	31 (28; 35)	31 (27; 35)	0.79
Pre‐pregnancy BMI, kg/m^2^	22.4 (20.1; 26.4)	24.0 (20.98; 27.0)	0.29
Smoking during pregnancy	9 (7.6%)	4 (6.1%)	1.00
Multiparous	51 (42.9%)	28 (43.1%)	0.98
Pre‐existing pathology			
Chronic hypertension	0 (0.0%)	3 (4.6%)	NA
Diabetes	0 (0.0%)	0 (0.0%)	NA
Pathology during pregnancy			
Hypertensive disorder (gestational hypertension or pre‐eclampsia)	6 (5.0%)	4 (6.1%)	0.74
Gestational diabetes	14 (11.8%)	8 (12.3%)	0.91
Labor onset			
Induction of labor	96 (80.7%)	47 (72.3%)	0.19
Indication for labor induction			
Ruptured membranes	35 (29.4%)	21 (32.3%)	0.17
Postdate pregnancy	16 (13.4%)	10 (15.4%)	
Suspected macrosomia	22 (18.5%)	7 (10.8%)	
Maternal pathology	8 (6.7%)	0 (0.0%)	
Maternal request	20 (16.8%)	13 (20.0%)	
Other indications	18 (15.1%)	14 (21.5%)	
Epidural analgesia	118 (99.2%)	65 (100.0%)	NA
Time from oxytocin onset to active first stage diagnosis, min	277 (185; 390)	180 (200; 364)	0.99
Cervical dilation at oxytocin discontinuation	7 (6; 9)	7 (6; 8)	0.16
Posterior or transverse presentation at active labor diagnosis	23 (19.5%)	25 (39.1%)	0.008
Mode of delivery			
Spontaneous	79 (66.4%)	44 (67.8%)	1.00
Instrumental vaginal delivery	28 (23.5%)	15 (23.1%)	
Cesarean delivery	12 (10.1%)	6 (9.2%)	
Neonatal morbidity at birth^a^	16 (13.5%)	2 (3%)	0.035

*Note*: Data are *n*/*N* (%) or median (IQR).

^a^ Neonatal morbidity at birth was assessed using a composite variable defined by an umbilical arterial pH at birth <7.10 and/or a base excess >10 mmol/L and/or umbilical arterial lactates >7 mmol/L and/or a 5‐minute Apgar score <7 and/or admission in neonatal intensive care unit.

## 4 DISCUSSION

Our results show that discontinuing oxytocin at the onset of the active phase of labor is associated with an improvement in fetal heart rate patterns. More than one‐third of women with initially abnormal tracings experienced normalization following discontinuation, characterized by improved variability and fewer decelerations. These changes occurred concomitantly with a significant reduction in uterine activity.

From a mechanistic perspective, the observed decrease in contraction frequency suggests that improved uteroplacental perfusion may contribute to FHR normalization. The association between oxytocin administration and uterine tachysystole is well established. In a large retrospective cohort study including 48 529 women, Heuser et al. reported an increased risk of tachysystole following oxytocin exposure (RR = 1.69; 95% CI 1.56–1.83; *p* < 0.0001 compared to non‐exposure) with a dose‐dependent relationship: each 5‐mIU/min increase in oxytocin dose was associated with a higher risk of tachysystole. Moreover, FHR abnormalities were significantly more frequent in the presence of tachysystole, affecting approximately one‐third of cases (RR = 1.89; 95% CI 1.65–2.17; *p* < 0.0001).^2^ These findings are consistent with impaired fetal oxygenation as an underlying mechanism. Indeed, Simpson et al. showed in 56 healthy nulliparous women undergoing labor induction with oxytocin that uterine tachysystole was associated with significant fetal oxygen desaturation. By stratifying contraction frequency, they showed a progressive decline in FSpO_2_ as contraction frequency increased. Specifically, FSpO_2_ decreased from 52.14% to 41.46% (*p* < 0.001) in women with 5 to <6 contractions per 10 min, and from 52.02% to 36.68% (*p* < 0.001) in those with ≥6 contractions per 10 min, accompanied by a higher frequency of non‐reassuring FHR patterns. This dose–response relationship further supports the association between uterine activity, fetal oxygenation, and FHR abnormalities.^3^

Consequently, several randomized trials have investigated oxytocin reduction strategies, including discontinuation at the onset of the active phase of labor.^6^, ^7^ The CONDISOX trial, a double‐blind multicenter randomized controlled trial including 1200 participants, reported a significantly lower risk of uterine hyperstimulation in the oxytocin discontinuation in the active phase group compared to the continuous oxytocin group (3.7% vs. 12.9%; *p* < 0.001). The risk of FHR abnormalities — defined as recurrent variable or late decelerations, tachycardia, bradycardia, minimal variability, or significant STAN events — was also significantly reduced (27.9% vs. 40.8%; *p* < 0.001).^6^

These findings are consistent with those of the STOPOXY trial, the parent study of our analysis, in which uterine tachysystole (a secondary outcome) was significantly less frequent in the discontinuation group than in the continuous group (6.3% vs. 10.4%).^7^ The absence of a detectable neonatal benefit despite improved fetal heart rate patterns likely reflects the low‐risk nature of the study population, the rarity of adverse neonatal outcomes, and the fact that FHR abnormalities are intermediate physiological markers rather than direct surrogates of neonatal morbidity. In addition, modern intrapartum surveillance and timely interventions may prevent fetal compromise from translating into measurable neonatal harm. Nevertheless, our findings highlight a crucial distinction in the high‐risk subgroup. Among women with initially abnormal tracings, we observed that FHR normalization following oxytocin discontinuation was significantly associated with reduced neonatal morbidity. This suggests that while the benefit may be diluted in the global population, the reversibility of FHR abnormalities constitutes a relevant prognostic factor for the fetus at highest risk.^7^

Most recently, a systematic review and meta‐analysis of fifteen randomized controlled trials confirmed that oxytocin discontinuation reduced the risk of uterine tachysystole (RR = 0.45; 95% CI 0.34–0.60) and non‐reassuring fetal heart rate tracing (RR = 0.64; 95% CI 0.49–0.82), but did not provide a detailed characterization of FHR patterns or uterine contractility. Our findings complement this literature by offering a precise physiological description of the immediate effects of oxytocin discontinuation on FHR characteristics and uterine activity.

Regarding clinical implications, while previous trials showed no reduction in overall operative delivery rates,^6^, ^7^ the normalization of initially abnormal FHR patterns in 35.3% (65/184) of cases has a direct impact on intrapartum management. In standard practice, a persistently abnormal tracing mandates heightened surveillance and triggers a cascade of interventions — including maternal repositioning, fluid bolus, or fetal scalp blood sampling — each carrying its own risks and resource implications. At the population level, the significant reduction in decelerations (64.1% vs. 48.6%) represents a clinically meaningful decrease in the number of women exposed to such intervention cascades. By rapidly restoring a reassuring pattern in over one‐third of high‐risk cases, oxytocin discontinuation constitutes a simple, non‐invasive first‐line approach. The marginal prolongation of the active phase of approximately 47 min reported in the STOPOXY trial^7^ represents a clinically acceptable trade‐off in this context.

The main strength of this pre‐specified secondary analysis is the detailed evaluation of physiological changes based on a comprehensive analysis of FHR parameters and uterine contraction frequency rather than a simple global classification. This approach is reinforced by the paired before‐and‐after design, in which each participant served as her own control, thereby minimizing the impact of inter‐subject variability. Furthermore, the use of high‐quality prospective data from a multicenter randomized trial and a blinded interpretation of FHR tracings by independent obstetricians unaware of the delivery outcomes enhances the robustness of the findings.

Several limitations should be acknowledged. First, regarding external validity, analyzable CTG data were only retrieved from six of the twenty‐one participating centers. However, these centers accounted for 56% of the total STOPOXY population, mitigating the risk of selection bias. Second, the analysis focused on a relatively short time window (1 h before and after discontinuation), capturing immediate physiological responses but potentially missing delayed effects. Third, signal quality was occasionally suboptimal, though uninterpretable tracings were strictly excluded. Fourth, we were unable to perform a comparable before‐and‐after analysis in the continuous oxytocin group. This limitation reflects two cumulative constraints: the absence of a clinically meaningful anchor event in this arm — randomization not corresponding to any change in management — and the inherent heterogeneity of oxytocin management in the continuous group, where clinicians could increase, maintain, or discontinue the infusion at their discretion in response to emerging FHR abnormalities. This variability in actual exposure precludes meaningful interpretation of any temporal FHR trend within this group. Indirect evidence from between‐group comparisons, however, suggests that the trajectory in the absence of discontinuation is towards FHR deterioration rather than improvement, as reflected by the higher rates of uterine tachysystole in the continuous arm of the STOPOXY trial (10.4% vs. 6.3%) and the higher rate of non‐reassuring FHR patterns in the CONDISOX trial (40.8% vs. 27.9%). Fifth, evaluators were not blinded to the exposure: the exact timestamp of oxytocin discontinuation was manually identified on each CTG recording to define the before and after periods, and the two consecutive windows were analyzed sequentially. This introduces a potential interpretation bias, as evaluators may have unconsciously expected improvement in the post‐discontinuation period. However, several features of our design limit this risk. FHR classification was performed using the standardized FIGO criteria, which define discrete, objective categories for baseline rate, variability, and decelerations, substantially constraining subjective interpretation. Furthermore, evaluators were blinded to neonatal outcomes — the most clinically meaningful source of ascertainment bias — and assessments were performed independently by two obstetricians, providing an additional safeguard. Finally, our study population was drawn from a selected randomized‐trial population with no major FHR abnormalities at randomization, which may limit generalizability to higher‐risk populations.

## 5 CONCLUSION

Among women receiving oxytocin during early labor, oxytocin discontinuation at the onset of the active phase is associated with improved FHR patterns, particularly reduced decelerations and improved variability, and with reduced uterine activity, suggesting a lower risk of hyperstimulation. Future research should evaluate if these physiological improvements translate into meaningful differences in labor management and maternal experience.

## AUTHOR CONTRIBUTIONS

Lise Lafforgue and Charles Garabedian conceived the study and performed the analysis of all fetal heart rate tracings. Aude Girault provided the study data, reviewed the fetal heart rate analyses, and conducted the statistical analyses. Lise Lafforgue drafted the first version of the manuscript, which was subsequently critically revised by Charles Garabedian and Aude Girault. Loïc Sentilhes, Raoul Desbriere, Diane Korb, Tiphaine Barjat, and Camille Le Ray contributed to data interpretation, critically reviewed the manuscript, and approved the final version for publication. All authors accept responsibility for the integrity of the work as published.

## FUNDING INFORMATION

This secondary analysis received no specific funding. The parent STOPOXY trial was funded by the French Ministry of Health through a national public research grant and by the Département de la Recherche Clinique et du Développement de l'Assistance Publique‐Hôpitaux de Paris. The funding bodies awarded the grant following external peer review for scientific quality. They had no role in the study design, data collection, analysis, interpretation of the data, or writing of the manuscript.

## CONFLICT OF INTEREST STATEMENT

Lise Lafforgue, Aude Girault, Charles Garabedian, Camille Le RAY, Tiphaine Barjat, Diane KORB, Raoul DESBRIERE have no conflict of interest to declare. Loïc Sentilhes has carried out consultancy work and been a lecturer for Ferring Pharmaceuticals, GlaxoSmithKline, Bayer, Pfizer, and Organon, and has been a lecturer for Norgine in the previous 3 years.

## ETHICS STATEMENT

The STOPOXY trial was approved by the Agence Nationale de Sécurité du Médicament et des produits de santé (ANSM) (national agency for drug safety), July 25, 2019, by the Comité de Protection des Personnes (Committee for protection of persons involved in biomedical research), December 5, 2019, and by the Commission Nationale de l'Information et des Libertés (CNIL) (French data protection authority), registration number MR001. Written informed consent was obtained from all participants, including information about the potential use of their data for future secondary analyses. This secondary analysis did not require additional consent, as it involved only de‐identified data collected during the original trial.

## Data Availability

The data that support the findings of this study are available from the corresponding author upon reasonable request.
